# Inherited Lipodystrophy Associated With POLD1 and CAVIN1 Mutations: Two Cases From the Indian Subcontinent

**DOI:** 10.7759/cureus.86622

**Published:** 2025-06-23

**Authors:** Shanmugam Sreekumar, Subbiah Sridhar, Palaniappan Sreenivasan, Sengottaiyan Palanivel, Vijai Shankar Chidambara Manivasagam, Dhivya Shanmugam

**Affiliations:** 1 Department of Endocrinology, Madurai Medical College, Madurai, IND; 2 Department of Medical Gastroenterology, Madurai Medical College, Madurai, IND

**Keywords:** achalasia, caveolin, congenital generalized lipodystrophy type 4, hypertriglyceridemia, lipodystrophy, mandibular dysplasia with deafness progeroid syndrome, phlebomegaly, primary amenorrhea, progeroid syndromes, secondary diabetes

## Abstract

Lipodystrophies comprise a large, heterogeneous group of disorders characterized by generalized or partial fat loss, accompanied by metabolic complications, including insulin resistance, which may or may not be associated with diabetes. Inherited lipodystrophies are a rare subgroup of lipodystrophies characterized by diverse systemic manifestations, posing a diagnostic and therapeutic challenge to clinicians. Here, we report two rare cases of lipodystrophy syndromes: mandibular dysplasia with deafness, progeroid features, and lipodystrophy (MDPL) (Online Mendelian Inheritance in Man (OMIM) #615381) and congenital generalized lipodystrophy type 4 (CGL4) (OMIM #613327). The presenting complaints were delayed puberty and young-onset diabetes in the former case and delayed puberty and achalasia cardia in the latter case. The molecular diagnosis was confirmed by whole-exome sequencing.

## Introduction

Mandibular hypoplasia, deafness, progeroid features, and lipodystrophy (MDPL) syndrome is a rare, autosomal dominant, inherited form of lipodystrophy caused by a mutation in the *POLD1* gene, associated with multisystemic features [1]. Although it shares some features with congenital lipodystrophies, it is characterized by a constellation of unique clinical features. Congenital generalized lipodystrophy type 4 (CGL4) is a rare, autosomal recessive disorder caused by mutations in the *CAVIN1* gene [2] and is characterized by unique features such as gastrointestinal dysmotility, myopathy, and arrhythmias. The overlap of clinical features among lipodystrophy syndromes poses a diagnostic challenge to treating physicians. The age of onset, pattern of fat loss, presence of systemic clinical features, and findings from a dual-energy X-ray absorptiometry (DXA) scan can help identify specific subtypes of lipodystrophy, some of which may respond to targeted treatments. Reporting these rare forms of diabetes contributes to the global understanding of such conditions, as their management and long-term follow-up often differ from those of more common types of diabetes.

## Case presentation

Case 1

**Figure 1:**
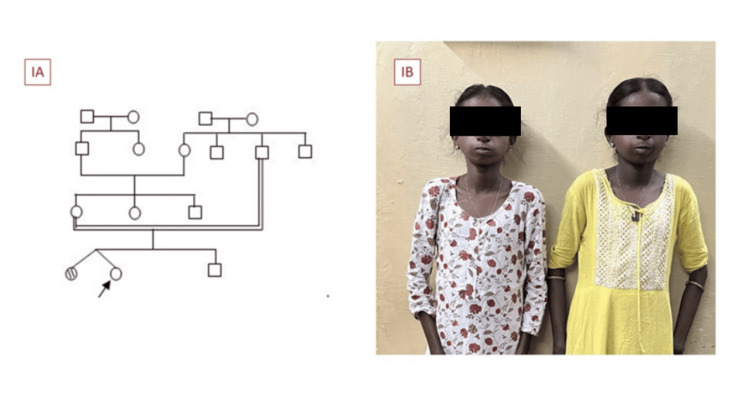
(A) Pedigree chart. (B) Family history of mandibular dysplasia with deafness progeroid (MDPL) syndrome

An 18-year-old female, born to third-degree consanguineous parents (Figure 1A) at full term with a birth weight of 2.3 kg, presented with underweight and primary amenorrhea. Moreover, she had a family history of a similar illness in a twin sibling (Figure 1B), and neither sibling underwent a previous evaluation. No similar illness was reported among other family members.

**Figure 2:**
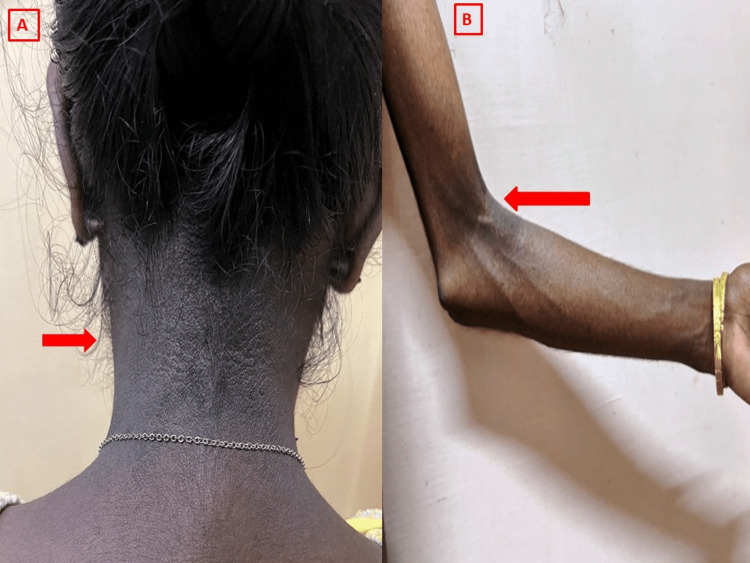
Clinical features of mandibular dysplasia with deafness progeroid syndrome. (A) Acanthosis nigricans. (B) Phlebomegaly

Physical examination revealed craniofacial dysmorphism, characterized by a triangular face with a progeroid appearance, a small chin with a hypoplastic mandible, a beaked nose, and prominent eyes. Despite significant subcutaneous fat loss over the face and limbs, and being underweight, she exhibited grade III acanthosis nigricans over the nape of the neck (Figure 2A). Additional findings included phlebomegaly (Figure 2B), elbow contractures, prominent musculature in the lower extremities, and hallux valgus.

The patient's anthropometric measurements were as follows: height 145 cm (-2.8 standard deviation score (SDS)), weight 25 kg (-4.69 SDS), and body mass index (BMI) 11.9 kg/m^2^ (-4.7 SDS). Tanner staging for sexual maturity revealed breast stage 1, pubic hair stage 3, absence of axillary hair, and clitoromegaly (clitoral index of 200 mm^2^; normal <35 mm^2^). Fasting and postprandial blood glucose levels were markedly elevated, with a significantly raised glycated hemoglobin (HbA1c) measured via high-performance liquid chromatography (HPLC). Despite a markedly low BMI, fasting insulin and C-peptide levels, measured by electrochemiluminescence immunoassay (ECLIA), were inappropriately elevated. The calculated homeostasis model assessment of insulin resistance (HOMA-IR) indicated severe insulin resistance. The lipid profile demonstrated hypertriglyceridemia with borderline total cholesterol and LDL cholesterol levels and reduced HDL cholesterol. Abdominal ultrasonography showed features suggestive of grade II hepatic steatosis.

Body fat percentage assessed using DXA in the arms, legs, and trunk was 10.8%, 7.3%, and 22.2%, respectively (normal mean values 32.0%, 38.5%, and 37.0%, respectively), and body fat percentage in the android and gynoid regions was 35.8% and 13.4%, respectively (normal 25.0%-30.0%). Hearing assessment revealed moderate, bilateral sensorineural hearing loss (SNHL). Whole-exome sequencing performed using peripheral blood DNA identified a heterozygous mutation (c3185A>G-p.Gln1062Arg) in exon 26 of the *POLD1* gene (chromosome 19) inherited as an autosomal dominant trait. This variant is classified as a likely pathogenic variant associated with MDPL syndrome (Online Mendelian Inheritance in Man (OMIM) #615381). The patient was started on a low-carbohydrate diet and basal-bolus insulin regimen with glargine and regular insulin, and metformin was added as an insulin-sensitizing agent. She was also treated with atorvastatin for dyslipidemia and a transdermal estrogen patch for pubertal induction.

Case 2

**Figure 3:**
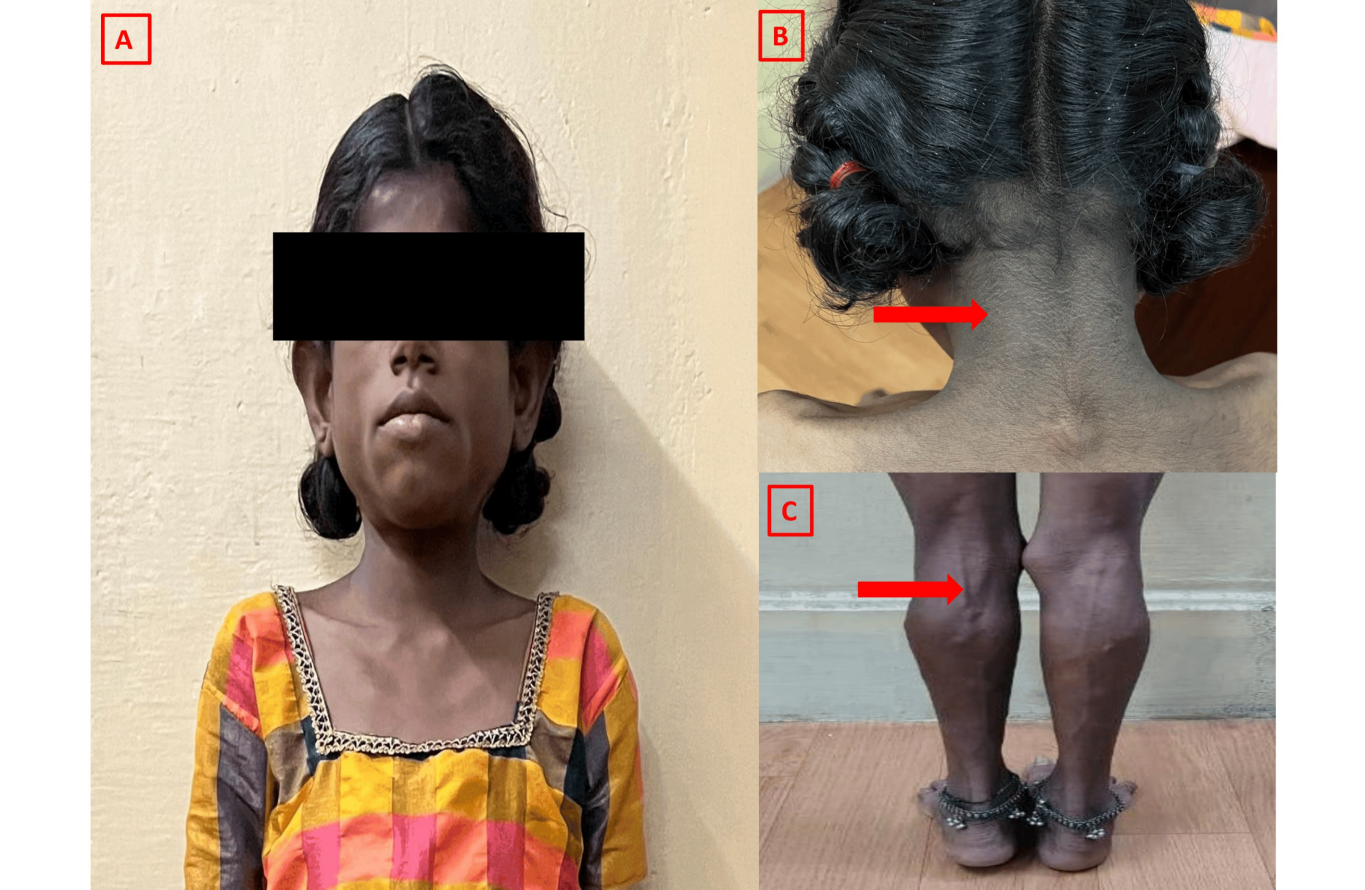
Clinical features of congenital generalized lipodystrophy type 4 showing acromegaloid facial appearance (A), acanthosis nigricans (B), and phlebomegaly (C)

A 15-year-old female, born to non-consanguineous parents, presented with underweight and delayed development of secondary sexual characteristics. She also reported a history of recurrent vomiting since childhood, which had not been previously evaluated. On physical examination, she exhibited acromegaloid facial features (Figure 3A) and grade III acanthosis nigricans over the neck (Figure 3B). There was a generalized absence of subcutaneous fat, prominent musculature in the lower limbs, and phlebomegaly in the lower extremities (Figure 3C).

The patient’s anthropometric measurements were as follows: height 136 cm (-4 SDS), weight 24 kg (-4.2 SDS), and BMI 12.9 kg/m^2^ (-4.2 SDS). Tanner staging for sexual maturity revealed breast stage 1, pubic hair stage 1, absence of axillary hair, and mild clitoromegaly (clitoral index of 50 mm^2;^ normal <35 mm^2^). Her fasting and postprandial blood glucose levels were within normal limits, with a corresponding HbA1c indicating optimal glycemic control. However, fasting insulin levels were elevated, suggesting underlying insulin resistance. The lipid profile revealed hypertriglyceridemia with normal total cholesterol and LDL cholesterol levels and borderline HDL cholesterol. Abdominal ultrasonography demonstrated hepatosplenomegaly.

**Figure 4:**
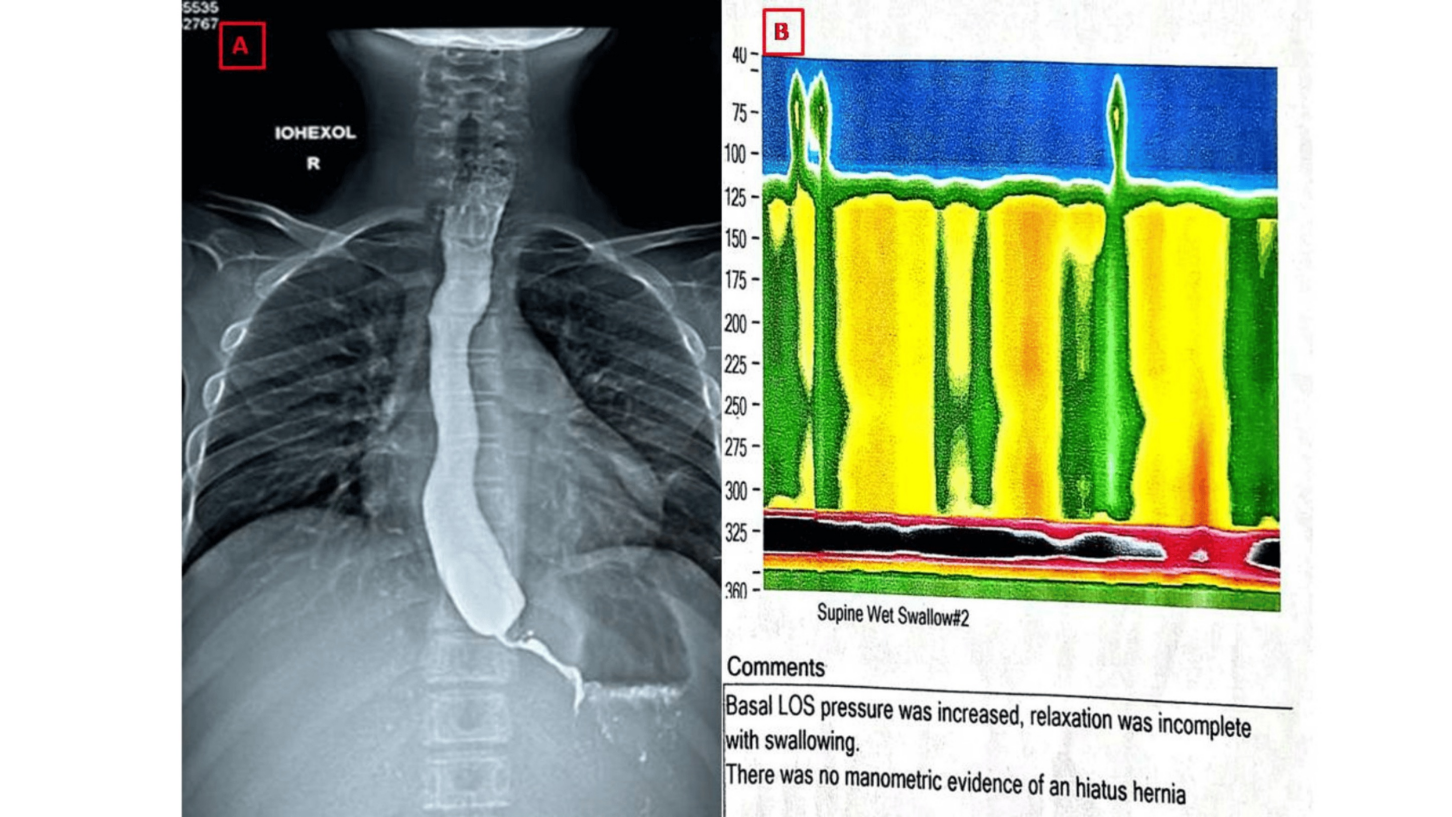
(A) Barium esophagogram with rat's tail appearance. (B) Esophageal manometry

The patient was examined by a medical gastroenterologist for recurrent vomiting. Barium esophagography revealed smooth narrowing of the distal esophagus with a characteristic “rat’s tail” appearance (Figure 4A) consistent with achalasia cardia. Esophageal manometry showed increased integrated relaxation pressure (90 mmHg; normal <15 mmHg) and pan-esophageal pressure (100 mmHg; normal <30 mmHg). Both these findings confirmed achalasia cardia type II (Figure 4B).

Esophageal gastroduodenoscopy indicated severe narrowing of the gastroesophageal junction, with the possibility of a motility disorder of the esophagus. The patient’s cardiac and audiometric evaluations were normal. Whole-exome sequencing identified a homozygous mutation (c311del p.Ser104ThrfsTer4) in exon 1 of the *CAVIN1* gene (chromosome 17) inherited as an autosomal recessive trait, which was likely a pathogenic variant associated with CGL4 (OMIM #613327). The patient underwent Heller’s cardiomyotomy for achalasia by a surgical gastroenterologist. She was advised dietary modifications and regular follow-up and was started on a transdermal estrogen patch for pubertal induction. Table 1 and Table 2 summarize the patient history, clinical features, biochemical and hormonal parameters, and genetic analysis. 

**Table 1:** Comparison of history, clinical features, and genetic analysis of case 1 (MDPL syndrome) and case 2 (CGL type 4) MDPL: mandibular hypoplasia, deafness, progeroid features, and lipodystrophy, CGL: congenital generalized lipodystrophy.

	Case 1	Case 2
History		
Presenting complaints	Underweight	Underweight
	Primary amenorrhea	Recurrent vomiting
Age at onset of symptoms	14 years	4 years
Consanguinity	Yes	No
Family history	Yes (twin sibling)	No
Clinical features		
Craniofacial dysmorphism	Yes	Yes
Distribution of fat loss	Arms and legs with relative sparing of trunk	Generalized
Acanthosis nigricans	Grade 3	Grade 3
Phlebomegaly	Yes	Yes
Clitoral index (normal < 35 mm^2^)	200 mm^2^	50 mm^2^
Hepatomegaly	Absent	Present
Auditory assessment	Bilateral sensory neural hearing loss	Normal
Genetic analysis		
Gene	DNA polymerase delta 1 catalytic subunit (POLD1)	Caveolae associated protein 1 (CAVIN1)
Chromosome	19q13.3-q13.4	17q21.2
Classification	Likely pathogenic/uncertain significance	Likely pathogenic

**Table 2:** Summary of biochemical and hormonal profiles of cases 1 and 2 HbA1c: glycated hemoglobin, HOMA-IR: homeostasis model assessment for insulin resistance, LH: luteinizing hormone, FSH: follicle-stimulating hormone.

Parameters	Case 1	Case 2	Normal range
Fasting plasma glucose (mg/dL)	228	109	70-99
Post-prandial plasma glucose (mg/dL)	287	136	140-199
HbA1c (%)	10.1	5.1	4-5.6
Fasting insulin (μIU/mL)	43.4	28.64	2.62-24.9
HOMA-IR	23.5	7.7	<2
C-Peptide (ng/mL)	3.2	1.7	0.81-3.85
Total cholesterol (mg/dL)	185	145	<200
Triglycerides (mg/dL)	387	220	<150
High-density lipoprotein (mg/dL)	41	45	>55
Low-density lipoprotein (mg/dL)	100	56	<100
LH (mIU/mL)	7.23	0.2	2.4-12.6
FSH (mIU/mL)	8.86	2.33	3.5-12.5
Prolactin (ng/mL)	19.8	9.6	4.04-15.2
Estradiol (pg/mL)	5	5.11	12.4-233
Testosterone (ng/dL)	32	18	2.9-40.8

## Discussion

Here, we report two cases of inherited lipodystrophy of different etiologies; both patients presented with underweight and features of severe insulin resistance. They were found to have *POLD1* and *CAVIN1* mutations and diagnosed with MDPL syndrome (OMIM #615381) and CGL4 (OMIM #613327), respectively. The prevalence of MDPL syndrome and CGL4 is estimated to be less than 1 in 1 million and 1 in 10 million, respectively. To date, only 29 cases of MDPL syndrome [1] and 30 cases of CGL4 [2] have been described in the literature. To the best of our knowledge, this is the third reported case of MDPL syndrome and the first reported case of CGL4 from the Indian subcontinent.

**Figure 5:**
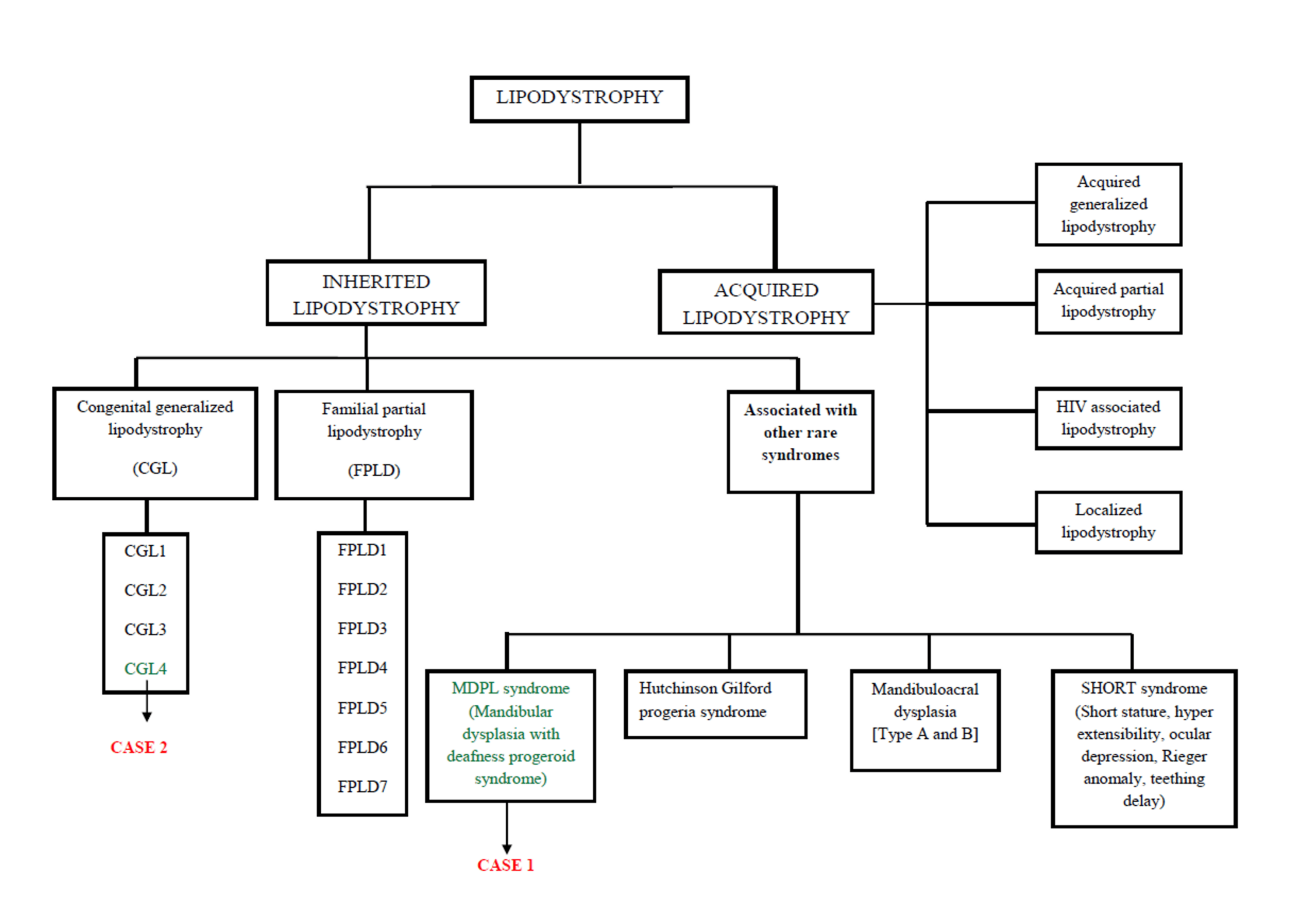
Summary of classification of inherited and acquired lipodystrophy

Lipodystrophies represent a group of rare disorders with selective deficiency of adipose tissue in the absence of nutritional deprivation and/or catabolic state [3]. Lipodystrophies may be classified into generalized, partial, localized, and syndromic types based on the distribution of adipose tissue loss and tissue of origin that affects the entire body or specific anatomical regions [4]. Figure 5 provides a summary of the classification of lipodystrophies.

Lipodystrophy should be suspected when any of the following clinical features are present: generalized or localized fat loss; severe acanthosis nigricans in lean individuals; disproportionately prominent biceps and calf muscles; phlebomegaly in the upper and lower limbs; and characteristic facies such as cushingoid, acromegaloid, or progeroid facies. The other features consistent with lipodystrophy are leanness with young-onset diabetes (<35 years of age), any syndromic diabetes with very high insulin (>2 units/kg/day) requirement [5], and diabetes with hepatic steatosis/transaminitis. The differential diagnoses of lipodystrophy are anorexia nervosa, acromegaly, hyperthyroidism, cancer cachexia, Donohue syndrome (leprechaunism), and Rabson-Mendenhall syndrome [4]. Lipodystrophy may mimic Cushing’s disease, often presenting as a pseudo-Cushing’s state, which may be observed in both congenital and acquired lipodystrophies. A notable example is the development of a pseudo-Cushing’s state following highly active antiretroviral therapy (HAART), a commonly encountered clinical scenario [6]. 

MDPL syndrome is caused by a heterozygous mutation in the *POLD1* gene inherited in an autosomal dominant manner. Loss of function mutation in the *POLD1* gene results in genomic instability and DNA damage. MDPL syndrome should be suspected in the presence of mandibular hypoplasia, bird-like facies, dental overcrowding, deafness, and joint contractures. Bird-like facies in MDPL syndrome are observed owing to the absence of facial subcutaneous fat, which leads to the prominent bony and muscular appearance of the face. Because of their normal phenotypical appearance, weight, and height at birth, patients are typically diagnosed at adolescence when they are examined for primary amenorrhea and diabetes mellitus. The progressive fat loss typically occurs by late childhood or at the end of the first decade of life. Our patient with MDPL syndrome had generalized fat loss, mandibular hypoplasia, phlebomegaly of arms, dysmorphic facies, acanthosis nigricans, and hearing loss.

Hypogonadism is reported in both males and females with lipodystrophy [7]. The precise prevalence remains unknown owing to the scarcity of published data. The various clinical manifestations of hypogonadism are delayed puberty and primary amenorrhea in females. The potential mechanism of delayed puberty is hyperandrogenism secondary to hyperinsulinemia. Other possible mechanisms are hypoleptinemia and hypothalamo-pituitary axis defect [8]. Oral estrogens are relatively contraindicated for pubertal induction owing to the risk of pancreatitis because of underlying hypertriglyceridemia [9]. Although oral, combined estrogen and progestin pills are known to negatively affect triglyceride and HDL cholesterol levels, data on their safety in lipodystrophy are lacking. Therefore, we started the patient on a transdermal estrogen patch for pubertal induction [9].

The onset of diabetes in lipodystrophy varies from adolescence to adulthood [10]. The potential mechanism is pancreatic dysfunction secondary to excessive circulating free fatty acids [10,11] and ectopic deposition of fat in the liver and muscles leading to insulin resistance. Insulin resistance due to adipocyte dysfunction and secondary ectopic lipid deposition is a hallmark of lipodystrophy. Typically, 80%-90% and 50%-70% of patients with generalized and partial lipodystrophy develop secondary diabetes mellitus, respectively. Our patient with MDPL (case 1) as well as her sister (twin) had diabetes mellitus at presentation.

CGL is classified into four types (CGL1-4) according to the mutation in genes involved in adipogenesis or maintenance of adipose tissue. CGL4 is caused by a mutation in the *CAVIN1 *gene that encodes for polymerase I and transcript release factor (PTRF) or Cavin1 protein [12]. Patients with CGL4 exhibit a near-total absence of metabolic fat in the subcutaneous, abdominal, and thoracic regions, with preserved mechanical and bone marrow fat. CGL4 should be suspected in the presence of lipodystrophic features with myopathy, gastrointestinal dysmotility, skeletal abnormalities such as atlantoaxial instability and scoliosis, and arrhythmias. Our second case of CGL4 with *CAVIN1* mutation exhibited a generalized absence of fat, acanthosis nigricans, prominent facial musculature, pseudo-acromegaloid appearance, phlebomegaly in lower limbs, proximal myopathy, and achalasia cardia.

Achalasia cardia should be suspected when there is progressive difficulty in swallowing (initially solids, followed by liquids), regurgitation of undigested food, and non-cardiac chest pain [13]. The other endocrine diseases associated with achalasia cardia are Allgrove syndrome, autoimmune hypothyroidism, and type 1 diabetes mellitus [14]. The proposed mechanism underlying esophageal dysfunction and achalasia cardia is defective caveolin, leading to dysfunction of the interstitial cells of Cajal (ICC) resulting in the impaired production of nitric oxide-the main factor mediating the relaxation of the lower esophageal sphincter [12]. Heller’s cardiomyotomy is the procedure of choice for achalasia cardia.

The fat mass ratio (percent trunk fat divided by percent leg fat) derived from the DXA findings, with cut-off values of >1.7 in males and > 1.2 in females, is useful in identifying lipodystrophy-like phenotypes; however, it is not employed as a diagnostic criterion [15]. The fat mass ratio was 3.04 in MDPL syndrome, which suggested a lipodystrophy-like phenotype. DXA and whole-body magnetic resonance imaging may be used as an additional diagnostic tool to look for body fat distribution. In addition to diabetes mellitus, hypogonadism, delayed puberty, achalasia cardia, and arrhythmias, other systemic manifestations reported in lipodystrophy are fatty liver, dyslipidemia, polycystic ovarian syndrome, and premature atherosclerosis [12]. Therefore, vigilant observation is required throughout the lifespan of the patient [15].

At present, there is no curative treatment available for lipodystrophy. Therapeutic management focuses on the treatment of comorbid conditions that include fenofibrate and statins for dyslipidemia and metformin, insulin, and pioglitazone for diabetes mellitus. In addition to improving glucose and lipid metabolism [5], metformin improves menstrual function [10] and decreases insulin requirements by improving insulin resistance [4]. Thiazolidinediones improve glycemic control and hypertriglyceridemia [16]. Metreleptin, a leptin analog, is an FDA-approved drug for generalized lipodystrophy and is not approved in other forms of lipodystrophy [17]. Metreleptin decreases hyperphagia, improves glycemic and lipid parameters and hepatic steatosis, reduces cardiovascular risk, promotes normal pubertal progression, and improves fertility [18]. Achalasia cardia needs surgical correction. Cosmetic surgery and adipose tissue transplantation may be suggested [19]. Long-term data are needed to understand the natural history of individual lipodystrophy, assess genotype/phenotype correlations, and determine the management of metabolic and reproductive abnormalities and therapeutic options. Recent studies have indicated that altered expression of lipodystrophic genes may contribute to the pathogenesis of metabolic syndrome, insulin resistance, and diabetes mellitus. Emerging evidence suggests a potential association, warranting further investigation into the role of specific lipodystrophic gene expression patterns in type 2 diabetes mellitus with insulin resistance features [20]. 

## Conclusions

Owing to the varied clinical presentations of lipodystrophy, recognition of individual manifestations is of paramount importance in differentiating the subtypes of lipodystrophy. Whole-exome sequencing will provide more valuable insights for distinguishing the type of lipodystrophy. There is no cure for this condition, and management is mostly supportive care. There is a need for future research in lipodystrophy to explore the impact of early diagnosis, the role of lifestyle modifications, and targeted therapies.
